# The Agronomic Potential of the Invasive Brown Seaweed *Rugulopteryx okamurae*: Optimisation of Alginate, Mannitol, and Phlorotannin Extraction

**DOI:** 10.3390/plants13243539

**Published:** 2024-12-18

**Authors:** Miguel A. Rincón-Cervera, Irene de Burgos-Navarro, Tarik Chileh-Chelh, El-Hassan Belarbi, Miriam Álvarez-Corral, Minerva Carmona-Fernández, Mohamed Ezzaitouni, José L. Guil-Guerrero

**Affiliations:** 1 Food Technology Division, ceiA3, CIAMBITAL, University of Almeria, 04120 Almeria, Spain or marincer@inta.uchile.cl (M.A.R.-C.); irenedeburgosnavarro@gmail.com (I.d.B.-N.); chileh@hotmail.es (T.C.-C.); mcf401@ual.es (M.C.-F.); mohamedezzaitouni6@gmail.com (M.E.); 2 Institute of Nutrition and Food Technology, University of Chile, Macul, Santiago 7830490, Chile; 3 Engineering Chemistry Department, University of Almeria, 04120 Almeria, Spain; ebelarbi@ual.es; 4 Organic Chemistry Division, University of Almeria, 04120 Almeria, Spain; malvarez@ual.es

**Keywords:** biostimulants, response surface methodology, invasive seaweeds, chemicals from macroalgae, bioactive compounds extraction

## Abstract

*Rugulopteryx okamurae* is an invasive brown macroalga that has recently proliferated in the western Mediterranean Sea, causing significant environmental challenges. This alga, however, contains valuable bioactive compounds—alginate, mannitol, and phlorotannins—that can serve as biofertilizers to promote plant growth and aid in bioremediation of degraded or contaminated soils. This study focused on optimizing the extraction of these compounds from *R. okamurae*, transforming an ecological issue into a beneficial resource. Algae samples collected from the Spanish Mediterranean coast were processed through a randomized factorial response surface design. Extraction conditions varied by time, temperature, algae-to-solvent ratio, and ethanol-to-water ratio to determine optimal yields. The highest yields achieved were 29.4, 11.9, and 0.35 g/100 g for alginate, mannitol, and phlorotannin’s under extraction conditions of 6, 6, and 3 h; 58.8, 60.0, and 60.0 °C; and an algae:solvent ratio of 1:50, 2:45, and 1.40 g/mL, respectively. Characterization of the extracted sodium alginate using ^1^H-NMR, FTIR, and high-resolution electron microscopy confirmed its high purity and typical morphological features. This study highlights a sustainable approach to mitigating the invasive spread of *R. okamurae* while supporting soil health and sustainable agriculture. Harnessing this invasive species’ biofertilizer potential provides a dual solution, aiding marine ecosystem conservation and developing eco-friendly agricultural practices.

## 1. Introduction

The brown seaweed *Rugulopteryx okamurae* is a native macroalga from the northwestern Pacific Ocean rapidly spreading as an invasive species in the western Mediterranean Sea and the Azores Islands [1]. Considering the environmental threat associated with the invasive behaviour of *R. okamurae*, the search for useful industrial applications of such biomass is timely. In this sense, there is growing evidence about the potential of this seaweed as biomass for anaerobic co-digestion, production of biofertilizers, and the development of bioplastic materials, together with potential applications in the pharmaceutical and food fields due to its anti-inflammatory and antimicrobial properties [1].

Brown seaweed belongs to the Phaeophyta phylum, a raw source of bioactive compounds. Among these, highlight phlorotannin’s, fucoxanthin, fucoidans, laminarin, mannitol, and alginates [2,3,4,5] (Supplementary Table S1). Phlorotannins are unique to brown seaweeds and are polyphenolic compounds with a crucial role in defense mechanisms against environmental stressors. They show promising applications in nutraceuticals, pharmaceuticals, and cosmetics due to their antioxidant, anti-diabetic, antimicrobial, and anti-inflammatory properties [6]. Phlorotannin acts as a defense mechanism against herbivores and protects brown seaweed from oxidative stress [7]. The presence of phlorotannins in brown seaweed contributes to their ecological fitness and resilience in harsh marine environments. Emerging evidence suggests a role for phlorotannin’s as plant growth promoters by increasing root surface area and plant biomass when used as biostimulants, and a potential use of these compounds to boost phytoremediation performance in soils contaminated by anthropogenic activities [8]. Fucoxanthin, a brown seaweed carotenoid, has gained attention for its anti-obesity and anti-inflammatory properties, and there are reports on its potential for promoting human health, thus, it is used in nutraceutical formulations [9]. Fucoidans are sulfated polysaccharides known for their diverse biological activities, including anti-inflammatory and anticoagulant properties, with potential in pharmaceutical research, particularly to develop novel therapeutic agents [10]. Laminarin is a storage polysaccharide found in brown algae currently investigated as a bioactive ingredient for food, cosmetic, and drug development due to its antioxidant, anti-tumor, anti-inflammatory, and neuroprotective potential [11]. Alginate, a polysaccharide derived from brown seaweed cell walls, is a complex linear polymer composed of guluronic and mannuronic acid residues that contribute to the structural integrity of the cell wall, providing flexibility and resistance to environmental stressors such as wave action and desiccation, acting as a growth stimulant for plants [12]. Beyond its ecological role, alginate has applications in various industries, including food, pharmaceuticals, and biotechnology, due to its gelling and stabilizing properties [13]. Mannitol, a sugar alcohol, is a predominant compound in brown algae. It constitutes a primary photosynthetic product and plays a central role in osmoregulation. Mannitol’s ability to act as a compatible solute allows brown seaweeds to thrive in fluctuating marine environments, including intertidal zones and estuaries. The role of mannitol in stress tolerance and carbon storage highlights its ecological importance in brown seaweed physiology [14]. Mannitol-containing biofertilizers act as protective in response to abiotic stressors, acting as chelating agents and, thus, improving the solubility of metals. Thereby, enhancing the uptake and translocation of metals by plants during phytoremediation [15].

Besides the potential of bioactive compounds of brown seaweeds as functional ingredients in food, cosmetic or pharmaceutical formulations, many studies have highlighted the bio-stimulant properties of brown algae as growth promoters in plants [15,16]. The role of this material as a biofertilizer results in increased nutrient availability in the soil, which enhances nutrient uptake by plants. The use of brown seaweed as biofertilizer is consistent with sustainable agricultural practices. Unlike conventional products, seaweed-based fertilizers are derived from renewable marine resources, reducing the environmental footprint associated with synthetic inputs [17]. Furthermore, seaweed cultivation does not require arable land, making it a viable option for regions facing land scarcity.

Brown seaweed helps improve soil structure by increasing soil aggregation and water-holding capacity [18]. Moreover, the polysaccharides in seaweed serve as substrates for beneficial soil microbes, creating an environment conducive to microbial activity [19]. This symbiotic relationship positively influences nutrient cycling and plant-microbe interactions.

The current work aims to enhance the extraction of alginate, mannitol, and phlorotannins from *R. okamurae* by testing different experimental conditions of temperature, extraction time, and algae:solvent ratio through a random response surface design.

## 2. Results and Discussion

### 2.1. Extraction and Physicochemical Alginate Characterization

Sodium alginate is a water-soluble compound with many pharmaceutical and food applications due to its encapsulating and coagulant properties, besides its gelling capacity in the presence of calcium [20]. Although alginate can be extracted at temperatures up to 100 °C and for long times if high yields are required, it is recommended to reduce the extraction temperature and time to qualitatively preserve alginate’s molecular weight and favour the economic balance of the extraction process [21]. Gels formed by high molecular weight alginate possess increased elasticity, viscosity, and strength than those formed with low molecular weight alginate [22]. Slight alginate degradation was observed at extraction temperatures higher than 40 °C [23,24], showing that a careful balance between quantity and quality must be considered when selecting the extraction temperature of this compound. In the present work, extraction temperatures up to 60 °C were tested to minimize alginate depolymerization.

**Figure 1:**
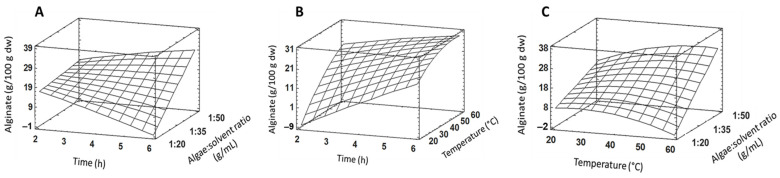
Response surface plots for alginate yield extraction from *R. okamurae* (g/100 g dw) as a function of time and algae: solvent ratio (**A**), time and temperature (**B**), and temperature and algae:solvent ratio (**C**).

Experimental results of alginate extraction from *R. okamurae* according to the 3^3^ response surface design in the current work are shown in Table 1, and response surface tridimensional graphs are depicted in Figure 1.

The second-order polynomial model fitting the response surface design for alginate extraction is:

Y = 14.028 − 1.286 Ti + 0.825 Te − 1.342 Ra − 0.167 Ti^2^ − 0.107 TiTe + 0.227 TiRa − 0.009 Te^2^ + 0.017 TeRa

where Y: dependent variable (alginate extraction yield); Ti: time; Te: temperature; Ra: algae/solvent ratio.

Based on the statistical analysis, the maximum predicted yield for alginate is 29.4 g/100 g dw when carrying out the extraction for 6 h, at 58.8 °C, and an algae:solvent ratio of 1:50 g/mL. That value is close to the amount of alginate previously reported in *R. okamurae* (32 g/100 g dw), which has been described as the main constituent of algal biomass [25]. Like our results, other authors found that 60 °C and 5 h were the optimal temperature and time to extract alginate from *Sargassum vulgare* [26].

Most studies have reported amounts of alginate in the 12–30 g/100 g dw range for brown seaweeds (Supplementary Table S1). Within the Order Dictyotales, the amount of alginate seems to be somehow higher in *R. okamurae* than in other species (Supplementary Table S1), which makes this invasive seaweed an excellent raw source for alginate isolation.

The analysis of variance (ANOVA) for alginate extraction is shown in Table 2. Seven effects were significantly different from zero (time, temperature, algae:solvent ratio, the interactions between time and temperature, time and algae:solvent ratio, temperature and algae:solvent ratio, and the quadratic effect of temperature). The F-value indicated that the temperature caused the highest variation in the alginate extraction, followed by the algae:solvent ratio. The R^2^ statistic showed that the fitted model explained 98.78% of the variability in alginate extraction. The adjusted R^2^ statistic, a more accurate indicator of the goodness of fit of multiple regression models, was 97.36%.

**Figure 2:**
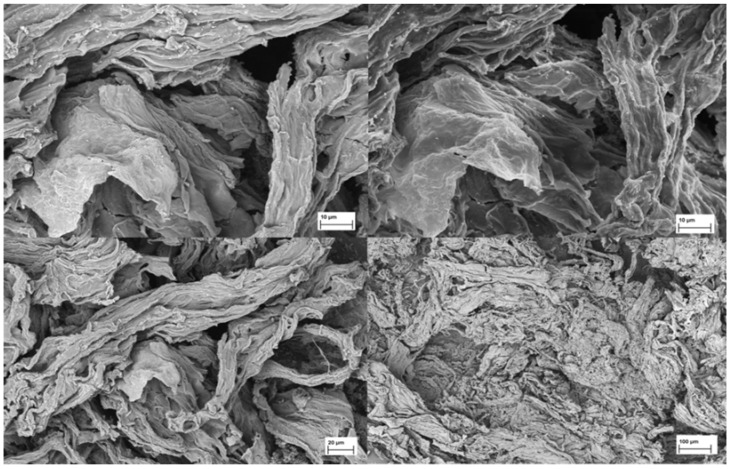
Images of purified and lyophilized sodium alginate obtained with a Field Emission Scanning Electron Microscope at different scales.

High-resolution FESEM images of sodium alginate extracted from *R. okamurae* are shown in Figure 2. Whereas commercial alginate shows a compact and smooth surface, a rough and less compact surface was observed in the extracted alginate [20,27], meaning that the capacity of water absorption of alginate from *R. okamurae* could be higher than that of commercial alginate, thus improving its gelling properties [20].

**Figure 3:**
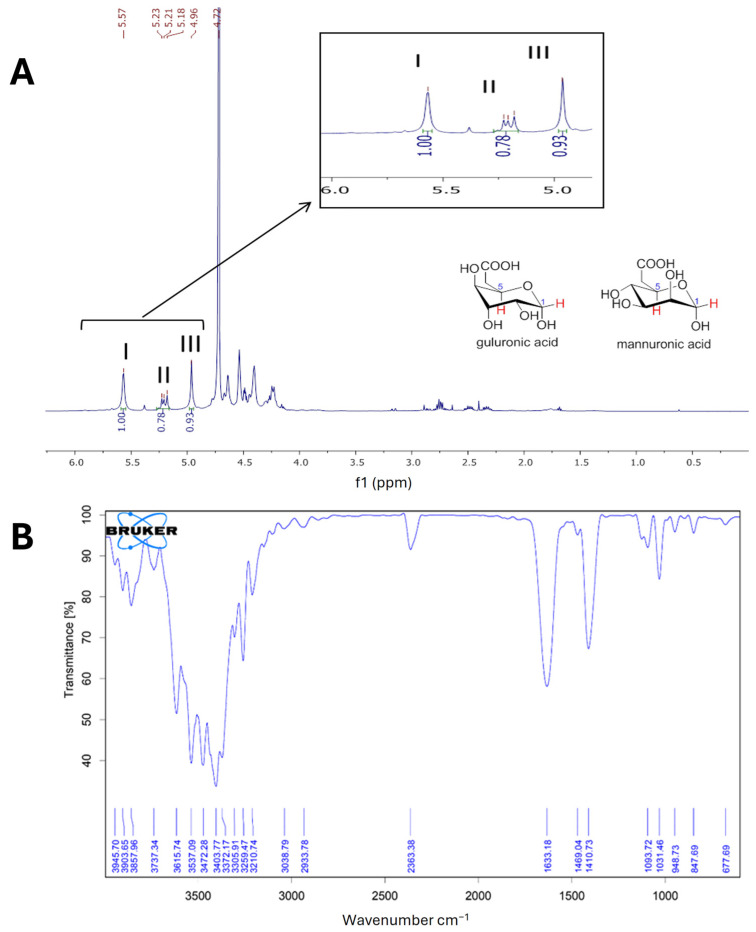
(**A**) ^1^H-NMR spectrum of sodium alginate extracted from *R. okamurae*. Signal I: guluronic acid anomeric proton (_HG1_); signal II: overlap of a mannuronic acid anomeric proton (H_M1_) and H5 of alternating blocks (_HGM5_); signal III: guluronic acid H5 proton in homopolymeric G blocks; (**B**) FT-IR spectrum of sodium alginate extracted from *R. okamurae*.

The alginate chain consists of units of α-L-guluronic acid (G) and β-D-mannuronic acid (M) linked via glycosidic bonds. The relative abundance of G and M in alginates contained in brown seaweed depends on the species and influences alginates’ physicochemical properties. The ^1^H-NMR spectrum of the extracted sodium alginate is shown in Figure 3A. Signals I (5.57 ppm), II (5.18–5.23 ppm), and III (4.96 ppm) are attributable to alginates; signal I is attributed to the anomeric proton of G; signal II is attributed to the overlapping of anomeric protons of M and the H-5 of alternating blocks (GM-5); and signal III is attributable to the H-5 proton of G residues in homopolymeric G blocks [28]. The estimation of the molar frequencies of G (F_G_) and M (F_M_) residues, the homopolymeric fractions (F_GG_ and F_MM_), the heteropolymeric fractions (F_GM_ or F_MG_), and the F_M_/F_G_ ratio in the purified alginate was carried out according to the equations proposed by [29], considering the intensity of the signals I, II and III in the ^1^H-NMR spectrum (Figure 3A):

F_G_ = I/(II + III) = 0.585

F_M_ = 1 − F_G_ = 0.415

F_GG_ = III/(II + III) = 0.544

F_MG_ = F_GM_ = F_G_ − F_GG_ = 0.041

F_MM_ = F_M_ − F_GM_ = 0.374

M/G = F_M_/F_G_ = 0.71

The resulting M/G ratio of 0.71 reveals a dominance of G over M. The homopolymeric fractions (F_MM_ and F_GG_) are remarkably high compared to heteropolymeric fractions (F_MG_ or F_GM_). Similar results have been recently reported in alginate extracted from *R. okamurae*, with F_G_ = 0.53, F_M_ = 0.47, and M/G = 0.88, whereas these authors found a M/G value for commercial alginate of 1.11 [20]. Other authors have reported M/G ratios lower than 1.0 in alginates extracted from *R. okamurae* [30].

Alginates with a higher proportion of G than M (M/G < 1) have higher viscosity and can form gels with higher resistance for food and cosmetic uses, and low F_MM_, intermediate F_GG_, and low values of heteropolymeric blocks promote the development of gel matrices [24]. The gelation capacity of alginate is calcium-dependent, and the structure of alginate gels has been described according to the “egg-box” model by the cross-linking of calcium cations with G residues [20,22].

The FTIR spectrum of purified alginate from *R. okamurae* shows typical absorption peaks of sodium alginate (Figure 3B). When compared with commercial sodium alginate (Sigma-Aldrich, Madrid, Spain, product No. 180947-500 G) previously published, the following signals can be assigned: the O-H stretching (3403.77 cm^−1^), COO- symmetric and asymmetric stretching (1633.18 and 1410.73 cm^−1^ respectively), and C-O-C stretching (1093.72 and 1031.46 cm^−1^ of M and G residues respectively) [27]. The peak at 847.69 cm^−1^ is attributed to the C1-H deformation vibration of M residues, and the signal at 948.73 cm^−1^ could be due to the C-O stretching vibration of uronic acid residues. Peaks in the 1230–1280 cm^−1^ region are related to S=O groups of sulphated polysaccharides such as fucoidan [28], and the absence of signals in that region reflects a high purity of the alginate extracted from *R. okamurae*.

The application of sodium alginate in agriculture has been highlighted to form biodegradable hydrogels with high water retention capacity, which is desirable when cultivated in degraded or arid soils [31]. Alginate can be used as a coating agent to encapsulate microbial formulations acting as plant growth promoters such as *Azospirillum brasilense*, *Azospirillum lipoferum*, *Glomus deserticola*, *Pseudomonas fluorescens*, or *Yarrowia lipolytica*, among others [32]. Inoculating plants with growth-promoting microorganisms offers an eco-friendly alternative to reduce the reliance on agrochemicals and rehabilitate contaminated soil, thus supporting sustainable agricultural practices.

### 2.2. Mannitol Extraction

**Figure 4:**
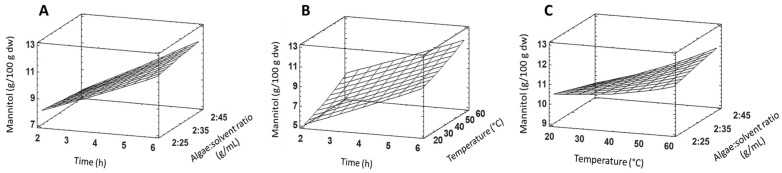
Response surface plots for mannitol extraction from *R. okamurae* (g/100 g dw) as a function of time and algae:solvent ratio (**A**), time and temperature (**B**), and temperature and algae:solvent ratio (**C**).

Experimental results of mannitol extraction in *R. okamurae* according to the response surface design (3^3^) are reported in Table 1, and response surface tridimensional graphs are depicted in Figure 4. Based on the statistical analysis, the maximum predicted amount for mannitol is 11.9 g/100 g dw when carrying out the extraction for 6 h, at 60 °C, and an algae:solvent ratio of 2:45 g/mL. The analysis of variance (ANOVA) results for mannitol extraction are shown in Table 2. Only time showed a significant effect regarding mannitol quantification (*p*-value = 0.0127). Considering the F-value, time caused the highest variation in the mannitol recovery. The R^2^ statistic showed that the fitted model explained 86.68% of the variability in mannitol quantification. The adjusted R^2^ statistic was 71.56%. The second-order polynomial model fitting the response surface design for mannitol is:

Y = 9.125 + 0.471 Ti − 0.047 Te − 0.155 Ra + 0.015 Ti^2^ − 0.002 TiTe + 0.013 TiRa + 0.0004 Te^2^ + 0.002 TeRa.

where Y: dependent variable (mannitol amount); Ti: time; Te: temperature; Ra: algae/solvent ratio.

Considering mean values of blocks 1 and 2 (Table 2), the highest amount of mannitol detected experimentally in this work was 11.7 g/100 g dw when the process was carried out at 60 °C, 6 h and using a 2:35 algae:solvent ratio, which is close to the maximum predicted by the model (11.9 g/100 g dw). Considering that time is the only variable with a significant influence in the process, it is reasonable to expect higher values of mannitol when the highest assayed time (6 h) was used regardless of the temperature values and algae:solvent ratio, as it was found experimentally. Among the results of mannitol in processes carried out at 6 h, the highest mean value was found at 60 °C, followed by 40 °C (10.8 g/100 g dw) and 20 °C (9.7 g/100 g dw), showing a small but not significant effect of temperature when processing the sample for mannitol quantification.

To our knowledge, the amount of mannitol in *R. okamurae* has not yet been reported. However, in other algae from the Dictyotales order the amount of mannitol is highly variable (Supplementary Table S1). For example, 0.4, 7.8, and 9.5 g mannitol/100 g dw have been reported for *Dictyota caribaea*, *Dictyota dichotoma*, and *Padina pavonica*, respectively [7,33]. Attempts to extract mannitol from *R. okamurae* biomass have been recently carried out, usually using a pretreatment step with further enzymatic hydrolysis, but the resulting amounts are rather low. For instance, 0.36 g mannitol/100 g dw was yielded after fermenting *R. okamurae* with *Aspergillus awamori* for 5 days followed by enzymatic hydrolysis for 24 h [34]. In contrast, other authors could not detect mannitol in *R. okamurae* after a hydrothermal acid treatment at 121 °C and further enzymatic hydrolysis [35].

Several studies have highlighted that mannitol has the potential to enhance plant growth, especially in situations of abiotic stress. In this sense, one work found that treating corn seeds with mannitol during a priming process significantly improved germination rates when exposed to water stress compared with untreated seeds [36]. Seed priming is a technique where seeds are exposed to specific moisture and temperature conditions to enhance germination and stress tolerance [37]. Additionally, the evidence suggests that applying mannitol to plant leaves can mitigate the harmful effects of heavy metals in plants growing on degraded soils. For example, some authors found that a mannitol solution (100 mM) applied on leaves of Cr-stressed wheat plants resulted in increased biomass, photosynthetic pigments, and antioxidant enzymes compared to plants without mannitol treatment [38]. However, it should be noted that while mannitol has shown beneficial effects in plants under stress conditions, it may have negative effects on unstressed plants in normal soil conditions [39]. In a study conducted by Habiba et al. [39], corn varieties exposed to Cr were subjected to foliar applications of mannitol (50 and 100 ppm), and plants showed enhanced growth, increased biomass, and higher levels of photosynthetic pigments. On the other hand, mannitol was found to decrease levels of reactive oxygen species (ROS) and Cr in leaves and roots. These results support the protective role of mannitol in plants grown in contaminated soils.

### 2.3. Phlorotannins Extraction

**Figure 5:**
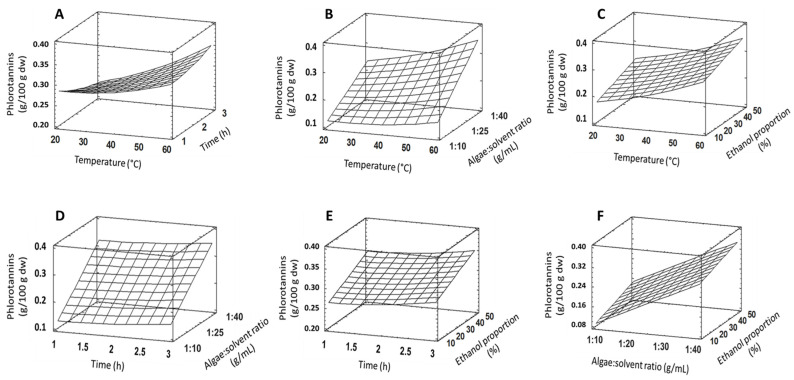
Response surface plots for phlorotannins extraction from *R. okamurae* (g/100 g dw) as a function of temperature and time (**A**), temperature and algae:solvent ratio (**B**), temperature and ethanol proportion in the extraction solvent (**C**), time and algae:solvent ratio (**D**), time and ethanol proportion in the extraction solvent (**E**), and algae:solvent ratio and ethanol proportion in the extraction solvent (**F**).

Experimental results of phlotorannins quantification in *R. okamurae* according to the response surface design (3^4^) are reported in Table 1, while response surface tridimensional plots are depicted in Figure 5. Based on the statistical analysis, the maximum predicted amount for phlorotannin is 0.35 g/100 g dw when carrying out the extraction for 3 h, at 60 °C, with an alga:solvent ratio of 1:40 g/mL, and an ethanol proportion of 50% in the extraction solvent. The results of an analysis of variance (ANOVA) for phlorotannin’s extraction are shown in Table 2. Four effects significantly differ from zero (algae:solvent ratio, time, ethanol proportion, and the interaction between time and algae:solvent ratio). Considering the F-value, the algae:solvent ratio caused the highest variation of phlorotannin yield. The R^2^ statistic showed that the fitted model explained 98.74% of the variability in phlorotannin’s quantification. The adjusted R^2^ statistic was 97.33%. The second-order polynomial model fitting the response surface design for phlorotannins is:

Y = 0.178 − 0.005 Te − 0.065 Ti + 0.003 Ra + 0.001 Et + 3·10^−5^ Te^2^ + 9·10^−4^ TeTi + 7·10^−5^ TeRa + 0.006 Ti^2^

where Y: dependent variable (phlorotannins amount); Ti: time; Te: temperature; Ra: algae/solvent ratio; Et: ethanol proportion in the extraction solvent.

The highest value of phlorotannins concentration obtained experimentally was 0.325 g/100 g dw (mean value of blocks 1 and 2) using 1 h of incubation at 60 °C with a 50% ethanol proportion and a 1:40 g/mL algae:solvent ratio (Table 1). This value was close to the maximum predicted amount of 0.35 g/100 g dw, but using only 1 h of incubation instead of 3 h, which means that incubation time could be decreased from 3 to 1 h without a large loss of phlorotannins. The detected amount of phlorotannins is within the range reported for these compounds in brown seaweeds (Supplementary Table S1).

Phlorotannins are a class of polyphenolic compounds biosynthesized by brown algae with functions analogous to condensed tannins in terrestrial plants [8]. Phlorotannins are products derived from polymerized phloroglucinol (1,3,5-trihydroxybenzene) residues. Besides the known bioactive properties of phlorotannins such as anticancer, anti-inflammatory, antioxidant, antidiabetic, anti-allergic, and UV protection, the use of these compounds as biostimulants has attracted increasing interest in recent years [40,41].

Soils contaminated with heavy metals promote the production of ROS, which are detrimental to plant health. Phlorotannins are antioxidants with scavenging activity against ROS in plants grown under stress conditions and have been described as stimulators of vegetative growth, plant metabolism and stress responses [8].

In a previous study, the efficacy of eckol, a phlorotannin, isolated from the brown kelp *Ecklonia maxima*, as a biostimulant was compared with commercial phloroglucinol and with Kelpak^®^ (Kelp Products (Pty) Ltd, Simon’s Town, South Africa), a commercial crop biostimulant derived from brown kelp, on corn seeds [42]. Seed priming was performed by soaking the seeds in solutions of eckol (10^−3^ to 10^−7^ M), phloroglucinol (10^−3^ to 10^−7^ M), or Kelpak^®^ (0.4%) for 18 h at room temperature. It was found that shoot length, root length and seedling weight were significantly increased with the eckol and phloroglucinol treatments compared with the use of Kelpak^®^, and eckol applied at a concentration of 10^−6^ M produced the highest number of germinal roots in corn seedlings than the other treatments, suggesting that this phlorotannin has a potential to promote plant growth and development.

## 3. Materials and Methods

### 3.1. Solvents and Reagents

Unless otherwise stated, all solvents and reagents used in this work are from Merck (Darmstadt, Germany).

### 3.2. Samples

Raw samples of *R. okamurae* were collected in December 2023 on the coast of Benalmadena (Malaga, Spain; geographic coordinates 36.580544, −4.539112) and delivered refrigerated (1 °C) to the laboratory. Samples were then washed with distilled water to remove salt, epiphytes, and sand, and placed in a forced air oven at 50 °C for 24 h. Once dried, samples were powdered and stored at 4 °C until analyses. The taxonomic identification of *R. okamurae* was confirmed by optical microscopy images (see Supplementary File S1).

### 3.3. Alginate Extraction

This procedure was accomplished following the protocol of Gomez et al. [43]. *R. okamurae* powder (2 g) was suspended in acidified distilled water (HCl 0.2 M) at different volumes and different extraction times and temperatures as specified in Section 3.7, and the mixture was then centrifuged (2000× *g*, 15 min at 4 °C). The resulting pellet was collected and extracted with 200 mL of a hot sodium carbonate aqueous solution (2% *w*/*v*) at 60 °C for 5 h under magnetic stirring. After the extraction and centrifugation (2.000× *g*, 15 min at 4 °C), sodium alginate was precipitated from the supernatant with 95% ethanol (1:2 *v*/*v*). The precipitate was washed sequentially with ethanol and acetone, and then dried in a forced air oven at 60 °C for 24 h. Alginate yield was reported as g/100 g dw algae.

### 3.4. Physicochemical Characterization of the Alginate Extracted from R. okamurae

Alginate was lyophilized as a preliminary step for physicochemical characterization. The morphology of the extracted alginate was determined using a high-resolution field emission scanning electron microscope (FESEM) Zeiss Sigma 300 VP (Oberkochen, Germany). The proton nuclear magnetic resonance (^1^H-NMR) spectrum of lyophilized alginate dissolved in D_2_O (50 g/L) was recorded in a Bruker Avance III 500 MHz equipment (Billerica, MA, USA) at 80 °C. The FTIR spectrum of the lyophilized alginate (1 mg in KBr pellet) was recorded in the range of 400–4000 cm^−1^ using Bruker Vertex 70 equipment (16 scans, 4 cm^−1^ resolution) at room temperature.

### 3.5. Mannitol Extraction

Mannitol was extracted according to the procedure of Cameron et al. [44]. *R. okamurae* powder (2 g) was suspended in acidified distilled water (HCl 0.2 M) at different volumes and different extraction times and temperatures as specified in Section 3.7, and the mixture was then centrifuged (2000× *g*, 15 min at 4 °C). To quantify the amount of mannitol in the supernatant, an aliquot of 5 mL was collected, and 5 mL of sulfuric acid 0.1 N and 5 mL of periodic acid 0.1 N were added. After 1 min, 2 g of potassium iodide and 20 mL of sulfuric acid 4 N were added. Then, a starch solution was added as an indicator and the mixture was titrated with a sodium thiosulfate 0.1 N solution until the colour turned from blue to off-white. Mannitol was quantified as g/100 g dw algae.

### 3.6. Phlorotannins Extraction

Phlorotannins were extracted from *R. okamurae* powder as described by Li et al. [45] with slight modifications. Briefly, alga powder (0.4 g) was dispersed in ethanol:water solution and incubated at different times and temperatures as specified in Section 3.7, under magnetic stirring (1200 g). After incubation, extracts were filtered with filter paper and the solid seaweed residue was washed with distilled water three times. Finally, the supernatant was used to determine total phlorotannins content (TPC) through the Folin-Ciocalteau method as described by Li et al. [45]. A calibration curve (0–200 µg/mL) with a phloroglucinol standard (Merck, Darmstadt, Germany) was used for quantification purposes, and results were reported as mg of phloroglucinol equivalents per 100 g of algal powder (mg PGE/100 g dw).

### 3.7. Statistical Analysis

An experimental random response surface design (3 levels for each assayed factor, 3^3^ for alginate and mannitol, and 3^4^ for total phlorotannins) adjusted to a quadratic model was used to obtain the most favorable values of the selected factors to yield the maximum values of alginate, mannitol, and phlorotannins from *R. okamurae* samples. The factors assessed in each case were: (i) time (2 to 6 h), temperature (20 to 60 °C), and algae:solvent ratio (1:20 to 1:50 g/mL) for alginate; (ii) time (2 to 6 h), temperature (20 to 60 °C), and algae:solvent ratio (2:25 to 2:45 g/mL) for mannitol; (iii) time (1 to 3 h), temperature (20 to 60 °C), algae:solvent ratio (1:10 to 1:40 g/mL), and ethanol proportion in the extraction solvent (10–50%, *v*/*v*) for phlorotannins. Experimental design and statistical analyses were performed using Statgraphics^©^ Centurion XVI, version 17.2.04 (StatPoint Technologies, Warrenton, VA, USA).

## 4. Conclusions

The need for sustainable practices in modern agriculture is imperative today, and efforts must be directed at increasing the quality and quantity of agricultural production in the context of a growing world population. However, the availability of arable land is decreasing due to soil pollution caused by human industrial activities. In this context, brown algae such as *R. okamurae* can become a natural, sustainable and environmentally friendly source of bioactive compounds with interesting agriculture applications, such as alginate, mannitol, and phlorotannins. In this work, it has been shown that *R. okamurae* is a potential source of such compounds since it contains them in concentrations located at the upper part of the range in which they are usually found in brown algae.

The F-value indicated that the temperature caused the highest variation in the alginate extraction, followed by the algae:solvent ratio; time caused the highest variation in the mannitol recovery; and the algae:solvent ratio caused the highest variation in phlorotannin’s yield. These compounds have a high potential to stimulate plant growth, especially in degraded soils. To complement the experimentation performed in this work, future research is needed to address challenges such as optimization of the extraction and purification methods by testing more variables, such as pH value and the ionic concentration of the extraction solvent; formulation and standardization; and large-scale production. The main drawback of the working method followed in this study is that the optimal values for the extraction variables were different for the compounds studied, especially concerning the algae:solvent ratio. More studies are also needed to explore the long-term effects of algal biostimulants on soil health and ecosystem sustainability.

## Figures and Tables

**Table 1:** Experimental results for the extraction of alginate (Alg), mannitol (Man), and phlorotannins (Phl) (g/100 g dw) from *R. okamurae* by using a 3-level response surface design with three independent variables for alginate and mannitol: time (Ti), temperature (Te) and algae:solvent ratio (Ra) (3^3^ design), and four independent variables for phlorotannins: time (Ti), temperature (Te), algae:solvent ratio (Ra), and ethanol proportion in the extracting solvent (Et).

Block	Ti (h)	Te (°C)	Ra (g/mL)	Algyield (g/100 g dw)	Ti (h)	Te (°C)	Ra (g/mL)	Man Yield (g/100 g dw)	Ti (h)	Te (°C)	Ra (g/mL)	Et (%)	Phl Yield (g/100 g dw)
1	4	60	1:20	7.8	6	20	2:45	8.3	2	20	1:25	50	0.208
1	2	60	1:50	19.5	6	40	2:25	11.0	3	20	1:40	30	0.223
1	6	60	1:35	14.1	2	40	2:35	7.1	1	60	1:40	50	0.331
1	6	20	1:50	17.0	2	60	2:45	8.2	3	60	1:25	10	0.188
1	2	20	1:20	8.0	6	60	2:35	11.0	1	20	1:10	10	0.100
1	6	40	1:20	6.7	4	40	2:45	8.7	1	40	1:25	30	0.181
1	4	40	1:50	18.2	4	60	2:25	8.8	2	40	1:40	10	0.222
1	2	40	1:35	12.1	2	20	2:25	7.0	2	60	1:10	30	0.106
1	4	20	1:35	7.9	4	20	2:35	7.5	3	40	1:10	50	0.117
2	4	60	1:20	8.6	6	20	2:45	11.1	2	20	1:25	50	0.185
2	2	60	1:50	20.5	6	40	2:25	10.5	3	20	1:40	30	0.192
2	6	60	1:35	13.7	2	40	2:35	6.4	1	60	1:40	50	0.319
2	6	20	1:50	19.1	2	60	2:45	7.0	3	60	1:25	10	0.193
2	2	20	1:20	8.5	6	60	2:35	12.3	1	20	1:10	10	0.111
2	6	40	1:20	6.6	4	40	2:45	8.0	1	40	1:25	30	0.186
2	4	40	1:50	20.0	4	60	2:25	10.5	2	40	1:40	10	0.220
2	2	40	1:35	13.5	2	20	2:25	6.9	2	60	1:10	30	0.100
2	4	20	1:35	6.1	4	20	2:35	8.5	3	40	1:10	50	0.133

**Table 2:** Analysis of variance (ANOVA) for alginate, mannitol, and phlorotannin’s extraction from *R. okamurae*.

Source	Sum of Squares	df	Mean Square	F-Value	*p*-Value
Alginate					
A: Time	10.584	1	10.584	14.36	0.0053
B: Temperature	57.037	1	57.037	77.41	0.0000
C: Algae/solvent ratio	37.453	1	37.453	50.83	0.0001
AA	0.762	1	0.762	1.03	0.3390
AB	18.347	1	18.347	24.90	0.0011
AC	34.850	1	34.850	47.30	0.0001
BB	23.363	1	23.363	31.71	0.0005
BC	18.875	1	18.875	25.62	0.0010
Blocks	1.561	1	1.561	2.12	0.1837
Total error	5.894	8	0.737		
Total (corr.)	474.363	17			
R^2^ = 98.78%; R^2^ adjusted for degrees of freedom (df) = 97.36%; Standard error of estimate = 0.8584; Mean absolute error = 0.4617.					
Mannitol					
A: Time	9.362	1	9.362	10.22	0.0127
B: Temperature	1.700	1	1.700	1.86	0.2103
C: Algae/solvent ratio	0.301	1	0.301	0.33	0.5824
AA	0.006	1	0.006	0.01	0.9384
AB	0.004	1	0.004	0.00	0.9462
AC	0.053	1	0.053	0.06	0.8154
BB	0.053	1	0.053	0.06	0.8168
BC	0.092	1	0.092	0.10	0.7596
Blocks	0.720	1	0.720	0.79	0.4012
Total error	7.330	8	0.916		
Total (corr.)	54.771	17			
R^2^ = 86.62%; R^2^ adjusted for degrees of freedom (df) = 71.56%; Standard error of estimate = 0.9572; Mean absolute error = 0.5778.					
Phlorotannin’s					
A: Time	0.0040	1	0.0040	32.51	0.0005
B: Temperature	0.0001	1	0.0001	1.00	0.3462
C: Algae/solvent ratio	0.0187	1	0.0187	153.71	0.0000
D: Ethanol proportion	0.0028	1	0.0028	23.04	0.0014
AA	0.0005	1	0.0005	4.34	0.0707
AB	0.0003	1	0.0003	2.66	0.1415
AC	0.0008	1	0.0008	6.73	0.0319
BB	0.00009	1	0.00009	0.76	0.4100
Blocks	0.00008	1	0.00008	0.62	0.4522
Total error	0.00097	8	0.00012		
Total (corr.)	0.0775	17			
R^2^ = 98.74%; R^2^ adjusted for degrees of freedom (df) = 97.33%; Standard error of estimate = 0.0110; Mean absolute error = 0.0062.					

## Data Availability

The authors confirm that the data supporting the findings of this study are available within the article and its Supplementary Materials.

## Supplementary Materials

The following supporting information can be downloaded at https://www.mdpi.com/article/10.3390/plants13243539/s1: Supplementary File S1. Supplementary Table S1. References [46,47,48,49,50,51,52,53,54,55,56,57,58,59,60,61,62,63,64,65,66,67,68,69,70,71,72] are cited in the Supplementary Materials.
