# Supplementary material for: The Agronomic Potential of the Invasive Brown Seaweed Rugulopteryx okamurae: Optimisation of Alginate, Mannitol, and Phlorotannin Extraction
Source: Plants (Basel). 2024 Dec 18;13(24):3539. doi: 10.3390/plants13243539 (PMC11677978; doi:10.3390/plants13243539)
Supplement: Supplementary file 1 [file plants-13-03539-s001.zip › Supplementary Table S1. Review on brown macroalgae.pdf]

Supplementary Material of the Article:

"The agronomic potential of the invasive brown seaweed *Rugulopteryx okamurae*: optimisation of alginate, mannitol, and phlorotannin extraction"

Supplementary Table S1. Phlorotannins, mannitol, and alginate contents of brown seaweed

Supplementary Table S1. Phlorotannins, mannitol, and alginate (g/100 g dw) of brown seaweed

Brown seaweed	Phlorotannins	Mannitol	Alginate	Reference
Order Chordales				
Chorda filum	3.1	8.0	15.5	[46,47,48]
Order Desmarestiales				
Desmarestia aculeata	3.9	5.8	12-16	[47,48,49]
Order Dictyotales				
Dictyota ciliolata	0.1	-	20.0	[50,51]
Dictyota dichotoma	0.1	-	21-23	[52,50]
Lobophora variegata	0.1-0.9	-		[50]
Padina boergesenii			~16.0	[51]
Padina gymnospora	0.1-0.6	-	16.0	[12,50]
Padina pavonica	-	9.5	13.0	[7,49]
Padina tetrastromatica	0.3	-	-	[53]
Rugulopteryx okamurae	0.2-0.3	-	32	[25,54]
Spatoglossum schroederi	4.3	-	-	[50]
Stypopodium zonale	1.7	-	-	[50]
Order Ectocarpales				
Chordaria flagelliformis	6.3	-	-	[48]
Dictyosiphon foeniculaceus	7.4	-	-	[48]
Ectocarpus siliculosus	5.8	-	-	[48]
Pylaiella littoralis	6.2	-	-	[48]
Order Fucales				
Ascophyllum nodosum	5.8	6.8-11.2	15-28	[5,47,55,56,57]
Bifurcaria bifurcata	3.7	-	16.0	[49,56]
Carpophyllum flexuosum	5.5	22.2	14	[58]
Carpophyllum plumosum	4.0	22.0	15.5	[58]
Cystoseira humilis	-	-	5.4-19.2	[12,59]
Cystoseira sedoides	-	-	-	[60]
Durvillaea antarctica	-	-	53	[61]
Durvillaea incurvata	0.5-3.1	-	-	[62]
Ericaria brachycarpa	-	-	12	[63]
Fucus serratus	4.27-18.1	11.2	18-28	[47,48,55,56,64]
Fucus spiralis	3.88	-	-	[56]
Fucus vesiculosus	1.2-23.2	8.1-16.4	6.0-22.0	[5,47,48,56,57,65,64]
Gongolaria barbata	-	-	19	[66]
Halidrys siliquosa	-	19.7	-	[47]
Himanthalia elongata	2.17-19.8	-	-	[56,64]
Hormophysa cuneiformis	-	-	13.3	[51]
Pelvetia canaliculata	3.39-8.2	-	-	[48,56]
Sargassum aquifolium	0.68	-	~18.0	[51,67]

Sargassum denticarpum	0.10	-	-	[67]
Sargassum mangarevense	-	13.0-14.0	7.7-11.3	[68]
Sargassum mcclurei	0.21	-	-	[67]
Sargassum oligocystum	0.24	-	-	[67]
Sargassum polycystum	0.07	-	17.1-27.6	[12,67]
Sargassum serratum	0.13	-	-	[67]
Treptacantha baccata	-	-	10.9	[69]
Turbinaria ornata	-	4.6-7.6	17.9-20.5	[68]
Turbinaria triquetra	-	-	22.2	[51]
Order Laminariales				
Ecklonia cava	3.3-3.4	20.1	20-38	[7,55,70]
Ecklonia kurome	3.0	-	-	[70]
Ecklonia radiata	0.1	30.7	13	[58]
Eisenia bicyclis	3.1	-	-	[70]
Laminaria digitata	0.13	6.6-20.0	22-52	[12,47,55,56,57,71]
Laminaria hyperborea	-	2-25	17-37	[55,57]
Lessonia spicata	0.3-3.2	-	-	[62]
Macrocystis pyrifera	-	-	18-33	[12,55]
Saccharina japonica	-	-	20-26	[55]
Saccharina latissima	-	3.7-19.0	17-33	[47,57,71]
Undaria pinnatifida	4.5	27.0	5.2-27.6	[46,57,58]
Order Sphacelariales				
Chaetopteris plumosa	2.6	-	-	[48]
Sphacelaria cirrosa	-	2.4	-	[47]

References

5. Ummat, V.; Sivagnanam, S.P.; Rameshkumar, S.; Pednekar, M.; Fitzpatrick, S.; Rai, D.K.; Padamati, R.B.; O'Donnell, C.; Tiwari, B.K. Sequential extraction of fucoidan, laminarin, mannitol, alginate and protein from brown macroalgae *Ascophyllum nodosum* and *Fucus vesiculosus*. Int. J. Biol. Macromol. 2024, 256, 128195. https://doi.org/10.1016/j.ijbiomac.2023.128195

7. Bogolitsyn, K.; Parshina, A.; Mamatmyrodov, K.; Polomarchuk, D.; Popov, N. Recent advances in biochemistry of marine phaeophyta: Chemical analysis, structural studies and applications. In *Studies in Natural Products Chemistry*; Atta-ur-Rahman, F.R.S., Ed.; Elsevier: Amsterdam, The Netherlands, **2023**; pp. 435–486. https://doi.org/10.1016/B978-0-443-18961-6.00007-X

12. Abka-Khajouei, R.; Tounsi, L.; Shahabi, N.; Patel, A.K.; Abdelkafi, S.; Michaud, P. Structures, properties and applications of alginates. Mar. Drugs **2022**, 20, 364. https://doi.org/10.3390/md20060364

25. Cebrián-Lloret, V.; Cartan-Moya, S.; Martínez-Sanz, M.; Gómez-Cortés, P.; Calvo, M.V.; López-Rubio, A.; Martínez-Abad, A. Characterization of the invasive macroalgae *Rugulopteryx okamurae* for potential biomass valorisation. Food Chem. **2024**, 440, 138241. https://doi.org/10.1016/j.foodchem.2023.138241

46. Kuda, T.; Taniguchi, E.; Nishizawa, M.; Araki, Y. Fate of water-soluble polysaccharides in dried Chorda filum a brown alga during water washing. J. Food Compos. Anal. **2002**, 15, 3–9. https://doi.org/10.1006/jfca.2001.1037

47. Olsson, J.; Toth, G.B.; Albers, E. Biochemical composition of red, green and brown seaweeds on the Swedish west coast. J. Appl. Phycol. **2020**, 32, 3305–3317. https://doi.org/10.1007/s10811-020-02145-w

48. Lemesheva, V.; Islamova, R.; Stepchenkova, E.; Shenfeld, A.; Birkemeyer, C.; Tarakhovskaya, E. Antibacterial, antifungal and algicidal activity of phlorotannins, as principal biologically active components of ten species of brown algae. Plants **2023**, 12, 821. https://doi.org/10.3390/plants12040821

49. Percival, E.; Young, M. Carbohydrates of the brown seaweeds: Part III. *Desmarestia aculeata*. Carbohydr. Res. **1974**, 32, 195–201. https://doi.org/10.1016/S0008-6215(00)82097-2

50. Ank, G.; Da Gama, B.A.P.; Pereira, R.C. Latitudinal variation in phlorotannin contents from Southwestern Atlantic brown seaweeds. PeerJ **2019**, 7, e7379. https://doi.org/10.7717/peerj.7379

51. Rashedy, S.H.; Abd El Hafez, M.A.E.; Dar, M.A.; Cotas, J.; Pereira, L. Evaluation and characterization of alginate extracted from brown seaweed collected in the Red Sea. Appl. Sci. **2021**, 11, 6290. https://doi.org/10.3390/app11146290

52. Deyab, M.A.; El-Katony, T.M.; El-Adl, M.F.; Ward, F.M. Temporal variation in chemical composition of *Dictyota dichotoma* (Hudson) J.V. Lamouroux (Dictyotales, Phaeophyceae) from Red Sea coast, Egypt. J. Coast. Life Med. **2017**, 5, 149–155. https://doi.org/10.12980/jclm.5.2017J7-3

53. Nair, D.; Vanuopadath, M.; Balasubramanian, A.; Iyer, A.; Ganesh, S.; Anil, A.N.; Vikraman, V.; Pillai, P.; Bose, C.; …; Nair, S.S. Phlorotannins from *Padina tetrastromatica*: Structural characterization and functional studies. J. Appl. Phycol. **2019**, 31, 3131–3141. https://link.springer.com/article/10.1007/s10811-019-01792-y

54. López-Hortas, L.; Flórez-Fernández, N.; Mazón, J.; Domínguez, H.; Torres, M.D. Relevance of drying treatment on the extraction of high valuable compounds from invasive brown seaweed *Rugulopteryx okamurae*. Algal Res. **2023**, 69, 102917. https://doi.org/10.1016/j.algal.2022.102917

55. Pérez, R. Ces algues qui nous entourent. In Conception Actuelle, Rôle Dans la Biosphère, Utilisation, Culture; Editions Quae: Versailles, France, **1997**.

56. Connan, S.; Goulard, F.; Stiger, V.; Deslandes, E.; Gall, E.A. Interspecific and temporal variation in phlorotannin levels in an assemblage of brown algae. Bot. Mar. **2004**, 47, 410–416. https://doi.org/10.1515/BOT.2004.057

57. Holdt, S.L.; Kraan, S. Bioactive compounds in seaweed: Functional food applications and legislation. J. Appl. Phycol. **2011**, 23, 543–597. https://doi.org/10.1007/s10811-010-9632-5

58. Zhang, R.; Yuen, A.K.L.; Nys, R.; Masters, A.F.; Maschmeyer, T. Step by step extraction of bio-actives from the brown seaweeds, *Carpophyllum flexuosum*, *Carpophyllum plumosum*, *Ecklonia radiata* and *Undaria pinnatifida*. Algal Res. **2020**, 52, 102092. https://doi.org/10.1016/j.algal.2020.102092

59. Belattmania, Z.; Kaidi, S.; El-Atouani, S.; Katif, C.; Bentiss, F.; Jama, C.; Reani, A.; Sabour, B.; Vasconcelos, V. Isolation and FTIR-ATR and 1H NMR characterization of alginates from the main alginophyte species of the Atlantic coast of Morocco. Molecules **2020**, 25, 4335. https://doi.org/10.3390/molecules25184335

60. Abdelhamid, A.; Jouini, M.; Amor, H.B.H.; Mzoughi, Z.; Dridi, M.; Said, R.B.; Bouraoui, A. Phytochemical analysis and evaluation of the antioxidant, anti-inflammatory, and antinociceptive potential of phlorotannin–rich fractions from three Mediterranean brown seaweeds. Mar. Biotechnol. **2018**, 20, 60–74. https://doi.org/10.1007/s10126-017-9787-z

61. Miller, I.J. Alginate composition of some New-Zealand brown seaweeds. Phytochemistry **1996**, 41, 1315–1317. https://doi.org/10.1016/0031-9422(95)00741-5

62. Erpel, F.; Mariotti-Celis, M.S.; Parada, J.; Pedreschi, F.; Pérez-Correa, J.R. Pressurized hot liquid extraction with 15% v/v glycerol-water as an effective environment-friendly process to obtain *Durvillaea incurvata* and *Lessonia spicata* phlorotannin extracts with antioxidant and antihyperglycemic potential. Antioxidants **2021**, 10, 1105. https://doi.org/10.3390/antiox10071105

63. Hachemi-Benmalek, N.; Nouani, A.; Benchabane, A. Valorization of brown algae (*Cystoseira caespitosa*) from local region in Algeria for sodium alginate extraction and their application in the immobilization of microbial pectinases. Alger. J. Environ. Sci. Technol. **2019**, 5, 1155–1162.

64. Heffernan, N.; Brunton, N.P.; FitzGerald, R.J.; Smyth, T.J. Profiling of the molecular weight and structural isomer abundance of macroalgae-derived phlorotannins. Mar. Drugs **2015**, 13, 509–528. https://doi.org/10.3390/md13010509

65. Creis, E.; Delage, L.; Charton, S.; Goulitquer, S.; Leblanc, C.; Potin, P.; Gall, E.A. Constitutive or inducible protective mechanisms against UVB radiation in the brown alga *Fucus vesiculosus*? A study of gene expression and phlorotannin content responses. PLoS ONE **2015**, 10, e0128003. https://doi.org/10.1371/journal.pone.0128003

66. Trica, B.; Delattre, C.; Gros, F.; Ursu, A.V.; Dobre, T.; Djelveh, G.; Michaud, P.; Oancea, F. Extraction and characterization of alginate from an edible brown seaweed (*Cystoseira barbata*) harvested in the Romanian Black Sea. Mar. Drugs **2019**, 17, 405. https://doi.org/10.3390/md17070405

67. Cuong, D.X.; Boi, V.N.; Van, T.T.T.; Hau, L.N. Effect of storage time on phlorotannin content and antioxidant activity of six *Sargassum* species from Nhatrang Bay, Vietnam. J. Appl. Phycol. **2016**, 28, 567–572. https://doi.org/10.1007/s10811-015-0600-y

68. Zubia, M.; Payri, C.; Deslandes, E. Alginate, mannitol, phenolic compounds and biological activities of two range-extending brown algae, *Sargassum mangarevense* and *Turbinaria ornata* (Phaeophyta: Fucales), from Tahiti (French Polynesia). J. Appl. Phycol. **2008**, 20, 1033–1043. https://doi.org/10.1007/s10811-007-9303-3

69. Khaya, K.; Raja, A.; Katif, C.; Bentiss, F.; Jama, C.; Reani, A.; Sabour, B.; Belattmania, Z. Chemical composition and antioxidant properties of *Treptacantha baccata* (Fucales, Ochrophyta) from the Atlantic coast of Morocco. Int. J. Algae **2022**, 24, 159–174. https://doi.org/10.1615/InterJAlgae.v24.i2.50

70. Shibata, T.; Kawaguchi, S.; Hama, Y.; Inagaki, M.; Yamaguchi, K.; Nakamura, T. Local and chemical distribution of phlorotannins in brown algae. J. Appl. Phycol. **2004**, 16, 291–296. https://doi.org/10.1023/B:JAPH.0000047781.24993.0a

71. Manns, D.; Deutschle, A.L.; Saake, B.; Meyer, A.S. Methodology for quantitative determination of the carbohydrate composition of brown seaweeds Laminariaceae. R. Soc. Chem. Adv. **2014**, 4, 25736. https://doi.org/10.1039/C4RA03537B
