# Supplementary material for: The Agronomic Potential of the Invasive Brown Seaweed Rugulopteryx okamurae: Optimisation of Alginate, Mannitol, and Phlorotannin Extraction
Source: Plants (Basel). 2024 Dec 18;13(24):3539. doi: 10.3390/plants13243539 (PMC11677978; doi:10.3390/plants13243539)
Supplement: Supplementary file 1 [file plants-13-03539-s001.zip › Supplementary File S1. Rugulopterix diferenciation.pdf]

Supplementary Material of the Article:

"The agronomic potential of the invasive brown seawwed *Rugulopteryx okamurae*: optimisation of alginate, mannitol, and phlorotannin extraction"

Supplementary File S1. Clues for the identification of *Rugulopteryx okamurae* and differentiation from *Dictyota dichotoma*

1. Samples

Samples were collected fresh, coming from seaweed arrivals. Samples of *R. okamurae* were also collected at dry state from algal carpets formed by arrivals, which were found accumulated and semi-buried in the sand of Los Lances beach (Cádiz, Spain). Thallus were separated from other supporting algae when needed. *R. okamurae* samples were differentiated from *Dictyota* species (Supplementary Figure 1) by examining their morphological and anatomical characteristics using both binocular and light microscopes, according to the procedures described by García-Gómez et al. [72]. Macroscopically, many individuals displayed numerous proliferous branchlets on both surfaces. All samples were labelled and frozen until analysis, which was delayed one week maximum. Adequate fresh sample (2 g) amount was placed in a forced air oven at 103 °C for 8 h for moisture determination. After this, the dry biomasses were ground to a fine powder and immediately analyzed. All results are shown on a dry weight (dw) basis.

2. Microscopic observations

This procedure was accomplished in freshly collected samples, and selected images are shown in Figure 1. The process involved the following steps: i) Dissection of algae in seawater; ii) Fixation with paraformaldehyde in 4% PBS (phosphate buffered saline) and pH=7.4 for 24 h at 4 ºC; iii) Washing, 3 times, 30 min each in PBS; iv) Dehydration in ethanol gradients: 50%, 70%, 90%, and 3x in 100% ethanol, 20 min in each step at room temperature; v) Infiltration, inclusion and polymerization in epoxy resin (EMbed 812); vi) Obtaining semi-thin sections of 1 μm thick stained with toluidine blue; and vii) Observation and photography of the sections of the algae.

Ultrathin serial sections were cut on a Reichert Ultracut S ultra microtome (Leica, Vienna, Austria) using a diamond knife, and the stained tissue sections were observed under a light microscope (Olympus, IX53, Tokyo, Japan).

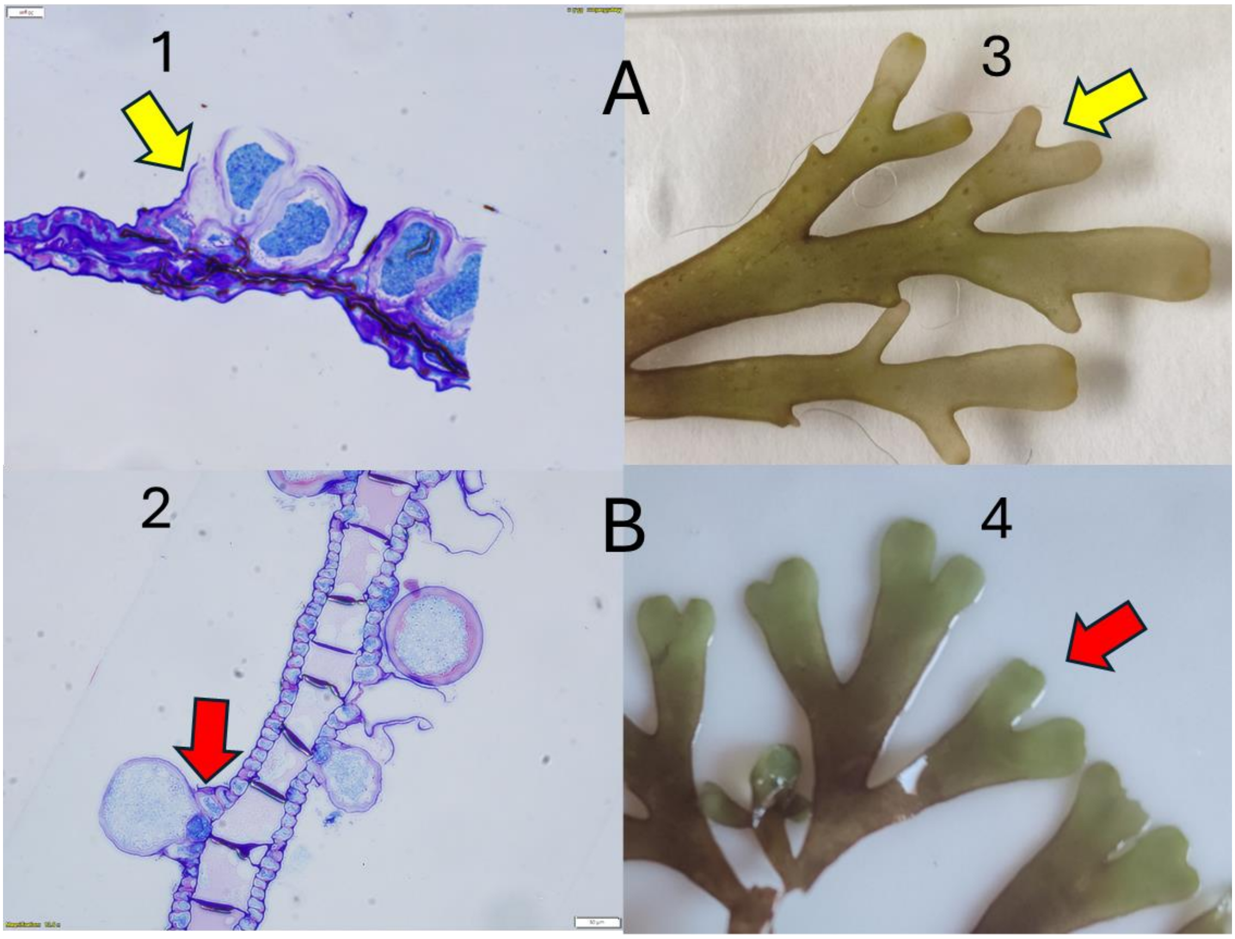

Supplementary Figure S1. The mature tetrasporangia has two stalk cells on the genus *Rugulopteryx* (A1), while it has only one in the genus *Dictyota* (B2). In *R. oka*murae, the dichotomous branching is non-isomorphic, and the apical margin of the thallus forms an obtuse angle in *R. okamurae* (A3), while it is isomorphic in *Dictyota*.

References:

72. García Gómez, J.C.; Sempere Valverde, J.; Ostalé Valriberas, E.; Martínez, M.; Olaya Ponzone, L.; Roi González, A.; Espinosa Torre, F.; Sánchez Moyano, J.E., Megina Martínez, C.; Parada, J.A. *Rugulopteryx okamurae* (EY Dawson) IK Hwang, WJ Lee & HS Kim (Dictyotales, Ochrophyta), alga exótica “explosiva” en el estrecho de Gibraltar. Observaciones preliminares de su distribución e impacto. *Almoraima. Revista de Estudios Campogibraltareños* **2018**, *49*, 97-113.
